# Suicide rates during and after the first COVID-19 lockdown in Germany in 2020

**DOI:** 10.1371/journal.pone.0289136

**Published:** 2023-09-01

**Authors:** Anne Elsner, Roland Mergl, Antje-Kathrin Allgaier, Ulrich Hegerl

**Affiliations:** 1 German Depression Foundation, Leipzig, Germany; 2 Universität der Bundeswehr München, Institute of Psychology, Neubiberg, Germany; 3 Department of Psychiatry, Psychosomatic Medicine and Psychotherapy, University Hospital Frankfurt, Goethe University Frankfurt (Distinguished Professorship funded by Dr. Senckenbergische Stiftung), Frankfurt am Main, Germany; Universidade Federal do Rio Grande do Sul, BRAZIL

## Abstract

The impact of the COVID-19 pandemic and of measures implemented to curb the spread of the virus on suicidal behavior has been investigated in different regions of the world, but does not yet allow to draw conclusions for Germany. Especially lockdowns might have effects on suicide rates via impact on mental disorders, changes in the choice of suicide method, a decrease in help seeking behavior, or a deterioration in the quality of medical care for people with mental disorders. The following research questions were addressed: i) did suicide rates in Germany in 2020 change during lockdown and non-lockdown periods when compared to a ten-year baseline? ii) was there a change in the proportion of suicide methods during the lockdown compared to baseline? An interrupted time-series analysis based on a linear regression was used. For the comparisons of predicted and observed suicide rates, excess suicide mortality rates (ESMR) were chosen among others. Changes in the choice of method were analyzed by comparing the rates of different methods to those at baseline. Although the mean suicide rate in 2020 was not significantly different from baseline, the weekly analysis of suicide rates revealed a significant difference (χ^2^ = 64.16; df = 39; p = 0.007), with some weeks showing higher and others lower rates than previous years. The effects for separate weeks were attenuated to non-significance after correction for multiple testing. Suicide mortality during the first lockdown in 2020 was significantly lower than expected (ESMR = 0.933; 95% CI: 0.890; 0.985) whereas, in the post-lockdown period, the registered suicide mortality was not significantly different from the expected one (ESMR = 1.024; 95% CI: 0.997; 1.051). During lockdown, there was a significant increase of the percentage of the suicide method categories jumping and ‘other methods’ and a decrease of poisoning and lying in front of a moving object. Being able to determine whether the choice of more or less lethal methods during lockdown versus non-lockdown periods partly explains this finding would require a representative assessment of attempted suicides.

## Introduction

The impact of the coronavirus disease 2019 and its counter-measures was widespread in all areas of life including mental health [1–3]. The prevalence of depression, anxiety, insomnia and PTSD among populations affected by COVID-19 were higher than in the general population [1]. Also, daily COVID-19 infection rates and reductions in human mobility were associated with increased prevalence of depression and anxiety [2]. According to a representative survey of 5,135 adults in Germany, in the year 2021, 49% of people with a life time diagnosis of depression (n = 1,038) reported a worsening of their depressive disorder because of anti-COVID-19 measures. This worsening comprised relapses (18%), increases in depression severity (20%), and the occurrence of suicidal ideations (9%). Increased bedtime, decreased physical activity, and unstructured days with increased ruminations were factors correlated with individual worsening of depression. Patients also reported reduced access to mental health care and a decline in the quality of medical care. These reports were also correlated with a worsening of depressive disorders [3]. Moreover, a decrease in consultations with a physician, e.g. for elderly people, was observed during the first lockdown in Germany [4].

Experts have cautioned that the effect of the COVID-19 pandemic and the restrictions to curb the spread of the virus on depression and other mental health disorders might lead to an increase of the rate of suicidal behavior [5, 6]. Indeed, multiple studies and meta-analyses have revealed a rise of suicide ideation, suicide attempts, and self-harm [7–9]. Additionally, physical and social distancing, especially during lockdown phases, may increase the risk of suicidal ideation not being noted by others and, therefore, reduce the likelihood of life-saving help being provided in time. Even doctors and therapists were consulted less frequently [10] and might thereby be less aware of increased suicidality in their patients.

Evidence on the impact of past pandemics or crises on suicidality suggests that, during previous epidemics, there was an increased risk of suicidal thoughts, behavior, and deaths [11]. Research concerning suicidality during the COVID-19 pandemic has indeed shown elevated levels of suicidal ideation compared to previous years, especially among younger people and vulnerable groups such as Hispanic persons, black persons, essential workers, unpaid caregivers for adults, and those receiving treatment for preexisting psychiatric conditions [8]. In a small study at a German university, the rate of students who reported suicidal ideation was twice as high in 2020 compared to previous years (9). In a meta-analysis that comprised 54 studies, a rise of suicide ideation, suicide attempts, and self-harm during the COVID-19 pandemic was found [7].

However, analyses of completed registered suicides do not suggest a rise in suicide rates, although many have cautioned about this possibility due to the increasing mental health problems. An analysis of preliminary suicide data for the first months of the pandemic stemming from several high- and upper-middle-income countries found no statistical evidence of a significant increase in suicide rates and in some countries evidence of a decrease compared with expected numbers derived from the preceding year [12]. Also, Wollschläger et al. found no systematically elevated suicide rates in the year 2020 compared to the years 2011–2019 in a region in Germany and a region in Italy [13]. Both regions were the same size but differently affected by the COVID-19 pandemic, with Italy recording five times more COVID-19 deaths and twice as many infections. In a systematic review of October 2020, no consistent evidence of a rise in the number of suicides around the world was found [14]. In Michigan, a decrease of suicide rates was found in 2020 after the onset of the COVID-19 pandemic compared to the previous years [15]. These studies would lead us to expect a decrease or no change in suicide rates.

On the other hand, there is evidence suggesting different effects of the pandemic depending on the time period studied. Studies conducted in Japan found an increase of suicide rates from July to October 2020 after an initial decrease. This finding was mainly driven by suicides of young women [16, 17]. In Mexico City, there was an increase in the number of monthly suicides during the first eight months of the pandemic with most of the increase occurring after June 2020. This finding was mainly driven by males [18]. Mitchell and Li found an overall decrease of suicide rates during the quarantine period in Connecticut. An excessive negative impact on racial minorities was reported, as a significantly higher proportion of suicides was registered in this group compared to the years before [19]. These findings of increasing suicide rates in certain time windows gave a hint towards a possible time factor.

When discussing suicidal behavior, one needs to keep in mind, that changes in the proportion of suicide methods can influence lethality of suicidal behavior. For example, when intoxication is chosen (survived in > 90%, lethality of 1.8% [20, 21]) instead of railway suicide or jumping from high buildings (lethality of 23% and 31.6% respectively [21]), the rate of attempted suicides might go up while the suicide rate goes down. Therefore, it is of interest whether measures taken against the pandemic induced a change in the choice of suicidal method. One study concerning admissions to psychiatric hospitals in Frankfurt/Main, Germany with around 765.000 inhabitants revealed that intoxications as a suicide attempt method increased and that more people attempted suicide in their home during the first ten months of the COVID-19 pandemic, while the rate of completed suicides remained unchanged [22].

In Germany, the first nationwide lockdown with far-reaching contact restrictions and social distancing regulations started in mid-March of 2020 and was loosened at the beginning of May with some measures such as keeping a minimum distance of 1.5 meters to others, restrictions of social contact for another month and prohibition of major events still in place (Lockdown period: 03/22/2020-05/06/2020). In the present study, we analyzed weekly suicide data. This allowed us to investigate the dynamics of suicide numbers in the timespan of the first lockdown and the timespan afterwards (period between the first and the second lockdown; 05/06/2020-12/16/2020). These were the most recent weekly data available in Germany at the end of 2022. Suicide data were analyzed using descriptive and exploratory statistics concerning the following research questions:

1. Do suicide rates in Germany since the start of the first lockdown until the end of 2020 (03/22/2020-12/31/2020) differ significantly from those during the same period of the previous ten years?
2. Do weekly suicide frequencies in Germany in 2020 differ significantly from the frequencies of the previous ten years?
3. Do suicide rates in Germany during the first lockdown (03/22/2020-05/06/2020) differ significantly from the rates of the previous ten years?
4. Do suicide rates in Germany between the first and second lockdown (05/06/2020-12/16/2020) differ significantly from the rates of the previous ten years?
5. Was there a change in the proportion of suicide methods during the first lockdown in 2020 compared to the previous ten years in Germany?

## Methods

### Data collection

The weekly number of suicides (ICD-10 codes X60-X84) by gender and age group for the period of 2010 to 2020 was obtained from the Causes of Death Statistics from the Research Data Centre of the Statistical Offices of the Federal States. These data are recorded officially and fully anonymized. Thus, a review by an ethics committee was not necessary. The data of the Research Data Centre are accessible for applying research institutions in exchange for a user fee. In Germany, suicides are ascertained by death certificates. The reporting lag is about one year. Population data by gender and age group were obtained via the publicly accessible genesis database of the Federal Statistical Office of Germany [23].

#### Methods used for suicide

In the Causes of Deaths Statistics, methods of suicidal acts are documented according to ICD-10 codes X60-X84. In the present analysis, methods are categorized into eight groups: poisoning (X60-X69), hanging (X70), drowning (X71), firearms (X72-X75), cutting by sharp objects (X78), jumping from high places (X80), lying in front of a moving object (X81, X82), and other methods (X76, X77, X79, X83, X84).

#### Definition of lockdown intervals

As Germany comprises 16 federal states, we decided for the time span when nationwide contact restrictions and social distancing regulations were in place that were valid for all states. The first lockdown was specified as the period from March 22 to May 6 of 2020, with Germany wide contact restrictions and social distancing regulations, followed by a relaxation of these regulations in most areas of life. The second so-called “hard lockdown” was defined to start on December 16 (as opposed to the “lockdown light” which started on November 2) and for reasons of current data availability could only be analyzed until December 31, 2020.

### Statistical analysis

In order to answer the research question of whether suicide rates during the first lockdown were significantly different from the expected suicide rates derived from the same period during the 10-year baseline (2010–2019), an interrupted time-series analysis was conducted. Suicide data from the lockdown period (03/22-05/06; years: 2010–2019) were selected to compute a predicted rate for the period of the first lockdown (03/22/2020-05/06/2020); next, predicted rates were compared with real suicide data of this period. For this purpose, a linear regression analysis was conducted with the annual suicide rate during the baseline period (2010–2019) as dependent variable and “year” as independent variable. Based on this regression model, predicted rates were computed for the COVID-19 period in 2020 as a whole and for the first lockdown period (see above) resulting in an expected suicide rate (ESR) for the first lockdown period in 2020 (according to the formula: ESR = (expected number of suicides (E) / population in the first lockdown period in 2020) * 100.000). Based on the latter formula, E was calculated.

In a next step, the excess suicide mortality rate (ESMR) was calculated as the quotient of the observed number of suicides (O) and E (ESMR = O/E). The 95% confidence interval (CI) of the ESMR was calculated according to the formula: ESMR ± 1.96 * standard error (se, with se = (square root of O) / E). The number of excess deaths by suicide (ED, defined as the difference between O in a certain period and E in the same period) was considered as well. The corresponding 95% CI was calculated by computing the 95% CI for O according to the formula: O ± (1.96 * (square root of O)) and subtracting E in a subsequent step. These analyses were conducted for the total number of suicides as well as specific numbers of suicides (e.g., gender-specific suicide data).

Analogously, we investigated whether suicide rates between the first and second lockdown in 2020 (05/06-12/16) were significantly different from the expected suicide rates derived from the same period during the 10-year baseline (2010–2019).

For weekly comparisons of registered suicide frequencies with expected suicide frequencies derived from a 10-year baseline chi-square goodness-of-fit tests were calculated. In addition to the global p value of this test, p values for each week were considered in order to identify weeks in 2020 during which the observed suicide frequencies were significantly higher or lower than the expected ones. For this purpose, an online calculator was used [24]. In this context, alpha-adjusted significance levels following the Bonferroni-Holm method were applied. We have abstained from computing suicide rates for the analysis of weekly suicide data in view of the fact that there had not been pronounced changes of the population number in Germany in 2020 and weekly suicide rates would have been very low.

In order to address the question of whether the proportion of more lethal suicide methods (like hanging) among all suicides in the total population during the first lockdown in 2020 (weeks 13–19) was significantly different from the corresponding proportion in the baseline period (2000–2019; weeks 13–19), chi-square tests for two-by-two tables were chosen with the rows representing the suicide methods (e.g., hanging and all other suicide methods) and the columns representing the period (baseline versus first lockdown).

SPSS version 26.0 was selected for the statistical analyses and the significance level α = 0.05 was chosen. All statistical tests were two-sided.

## Results

### Excess suicide mortality rates during the COVID-19 period in 2020

During the COVID-19 period in 2020 (weeks 13–52), 7,041 persons died from suicide in Germany and 6,991 persons were expected to die based on findings for this period during the years 2010–2019. The total excess suicide mortality rate (ESMR) was 1.0071 (95% CI: 0.9836; 1.0306). Thus, the registered suicide mortality did not significantly differ from the expected suicide mortality. In line with this result, the excess deaths from suicide (50; 95% CI: -114.88; 214.04) were not significantly different from zero.

### Weekly comparisons of the registered number of suicides in 2020 in Germany with the expected number derived from a ten-year baseline

**Fig 1:**
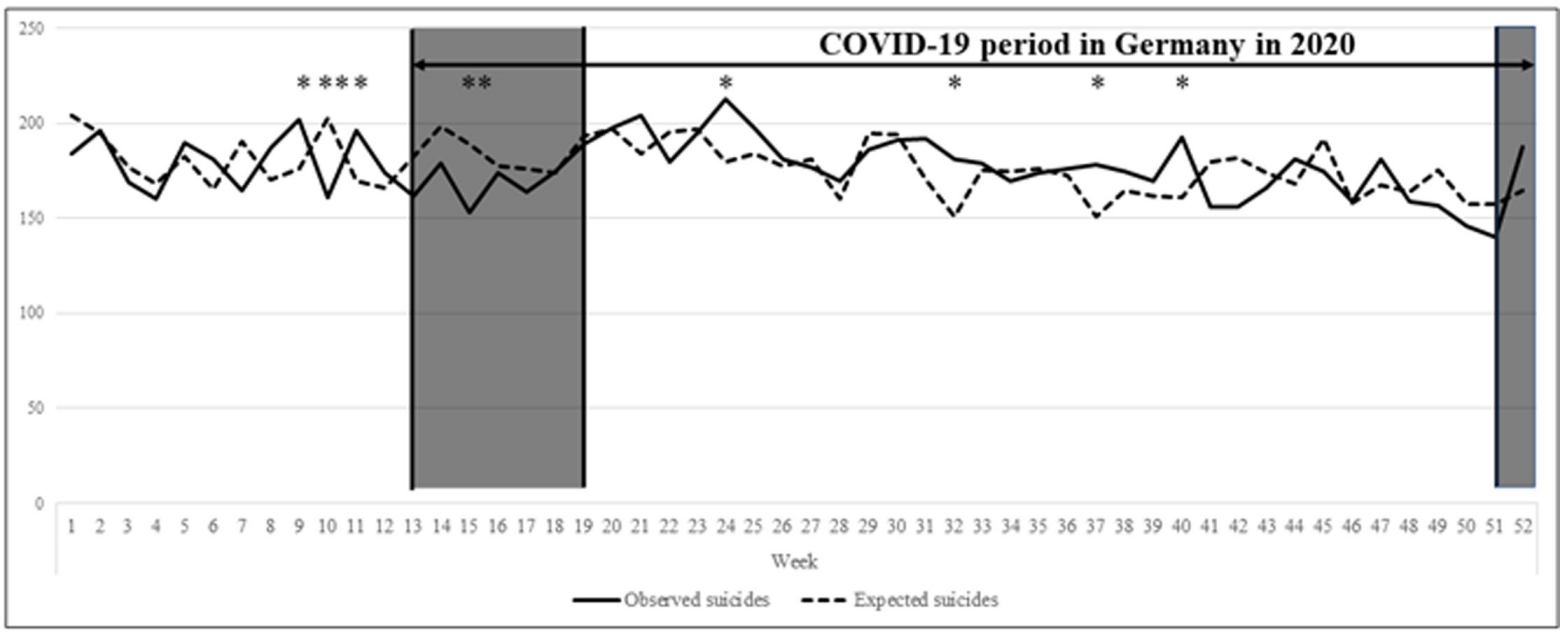
Registered and expected suicide frequencies per week in 2020. Asterisks indicate a p<0.05 before multiple testing. No difference is statistically significant after alpha-adjustment for multiple testing (α = 0.05/52 = 0.00096). For all p-values and suicide frequencies see Table 1. Black highlighting illustrates lockdown times. The predicted values for the total sample based on annual suicide rates for single weeks between 2010 and 2019. * p < 0.05; ** p < 0.01.

Overall, weekly registered suicide frequencies in Germany in 2020 were significantly different from the expected suicide frequencies derived from a ten-year baseline (χ^2^ = 90.42; df = 51; p < 0.001). Similar results were found for the COVID-19 period in 2020 in Germany (weeks 13–52: χ^2^ = 64.16; df = 39; p = 0.007). Post-hoc analyses for single weeks revealed that the number of suicides was significantly higher than expected in weeks 9 and 11 (before the first lockdown) and in weeks 24, 32, 37, and 40 (between the first and second lockdown). In contrast, the number of suicides was significantly lower than expected in weeks 10 (before the first lockdown) and 15 (during the first lockdown) (see asterisks in Fig 1). The aforementioned differences were no longer significant after correction for multiple testing. For a complete overview of suicide frequencies per week in 2020 see Table 1.

**Table 1:** Registered and expected suicide frequencies per week in 2020 in Germany.

Week	Registered number of suicides	Expected number of suicides	Difference	p value
**1**	184	204.49	-20.49	0.16
**2**	196	194.69	1.31	0.94
**3**	169	176.64	-7.64	0.54
**4**	160	168.33	-8.33	0.53
**5**	190	182.90	7.10	0.60
**6**	181	165.54	15.46	0.24
**7**	165	190.59	-25.59	0.06^+^
**8**	187	170.51	16.49	0.22
**9**	202	175.95	26.05	0.048*^a^
**10**	161	203.12	-42.12	0.003**^a^
**11**	196	169.84	26.16	0.044*^a^
**12**	174	166.08	7.92	0.53
**13**	162	182.72	-20.72	0.12
**14**	179	198.15	-19.15	0.17
**15**	153	188.87	-35.87	0.008**^a^
**16**	174	177.51	-3.51	0.76
**17**	164	176.44	-12.44	0.36
**18**	175	174	1	0.94
**19**	189	193.33	-4.33	0.77
**20**	198	196.99	1.01	0.94
**21**	204	184.13	19.87	0.14
**22**	180	195.61	-15.61	0.25
**23**	195	196.69	-1.69	0.89
**24**	213	179.91	33.09	0.013*^a^
**25**	198	184.14	13.86	0.30
**26**	181	177.41	3.59	0.76
**27**	177	180.94	-3.94	0.76
**28**	170	160.05	9.95	0.43
**29**	186	194.62	-8.62	0.52
**30**	191	194.21	-3.21	0.83
**31**	192	170.74	21.26	0.11
**32**	181	150.93	30.07	0.014*^a^
**33**	179	175.38	3.62	0.76
**34**	170	174.59	-4.59	0.70
**35**	174	175.97	-1.97	0.88
**36**	176	172.64	3.36	0.82
**37**	178	150.65	27.35	0.027*^a^
**38**	175	164.49	10.51	0.39
**39**	170	161.50	8.50	0.53
**40**	193	161.25	31.75	0.011*^a^
**41**	156	180.10	-24.10	0.07^+^
**42**	156	182.04	-26.04	0.052^+^
**43**	166	174.19	-8.19	0.54
**44**	181	168.59	12.41	0.35
**45**	175	192.17	-17.17	0.22
**46**	159	158.52	0.48	1
**47**	181	167.80	13.20	0.31
**48**	159	164	-5	0.69
**49**	157	175.76	-18.76	0.15
**50**	146	157.77	-11.77	0.34
**51**	140	157.80	-17.80	0.15
**52**	188	164.68	23.32	0.07^+^

n.s. = not significant

^+^ p < 0.10

* p < 0.05

** p < 0.01.

^a^ The difference is not statistically significant after alpha-adjustment for multiple testing (α = 0.05/52 = 0.00096).

The expected values for the total sample based on annual suicide rates for single weeks between 2010 and 2019.

### Excess suicide mortality rates during the first lockdown in 2020

In 2020, 1,196 individuals died from suicide during the first lockdown in Germany. The total ESMR was 0.9325 (95% CI: 0.8796; 0.9853, see Table 2). Thus, the registered suicide mortality was significantly lower than the expected one. In line with this finding, the excess deaths from suicide (-87; 95% CI: -154.38; -18.82, see Table 2) were significantly lower than zero. For results of subgroup analyses see Table 2.

**Table 2:** Excess suicide mortality rates during the first lockdown in Germany in 2020.

Group	Expected suicide rate	Observed suicide rate	Expected number of suicides	Observed number of suicides	ESMR (95% CI)	Excess number of suicides (95% CI)
**Total** ^**1**^	1.5424	1.4383	1282.60	1196	0.9325 (0.8796; 0.9853)	-86.60 (-154.38; -18.82)
**Men** ^**1**^	2.3382	2.1766	959.26	893	0.9309 (0.8699; 0.9920)	-66.26 (-124.83; -7.69)
**Women** ^**1**^	0.7631	0.7192	321.47	303	0.9425 (0.8364; 1.0487)	-18.47 (-52.59; 15.65)
**Age groups**	‐‐‐	‐‐‐	‐‐‐	‐‐‐	‐‐‐	‐‐‐
**≤24 years men** ^**1**^	0.4997	0.3793	51.38	39	0.7591 (0.5208; 0.9973)	-12.38 (-24.62; -0.14)
**≤24 years women** ^**1**^	0.1473	0.1143	14.17	11	0.7763 (0.3175; 1.235)	-3.17 (-9.67; 3.33)
**25–44 years men** ^**1**^	1.7106	1.6714	182.18	178	0.9771 (0.8335; 1.1206)	-4.18 (-30.33; 21.97)
**25–44 years women** ^**1**^	0.4638	0.4818	47.17	49	1.0388 (0.7479; 1.3297)	1.83 (-11.89; 15.55)
**45–64 years men** ^**1**^	2.7802	2.5265	335.63	305	0.9087 (0.8068; 1.0107)	-30.63 (-64.86; 3.60)
**45–64 years women** ^**1**^	1.0045	0.8687	121.41	105	0.8648 (0.6994; 1.0303)	-16.41 (-36.49; 3.67)
**≥65 years men** ^**1**^	4.8443	4.6245	388.64	371	0.9546 (0.8575; 1.0518)	-17.64 (-55.39; 20.11)
**≥65 years women** ^**1**^	1.3362	1.3465	136.95	138	1.0077 (0.8395; 1.1758)	1.05 (-21.98; 24.08)

CI = confidence interval; ESMR = excess suicide mortality rate.

^1^ Prediction from a regression analysis was based on annual suicide rates for the period of the first lockdown between 2010 and 2019.

### Excess suicide mortality rates between the first and second lockdown in 2020

In 2020, 5517 individuals died from suicide between the first and second lockdown in Germany. Total excess suicide mortality rate (ESMR) was 1.0239 (95% CI: 0.9968; 1.0509, see Table 3); thus, the registered suicide mortality was not significantly higher than the expected one. The excess deaths from suicide (129; 95% CI: -16.99; 274.17, see Table 3) did also not become statistically significant. For results of subgroup analyses see Table 3.

**Table 3:** Excess suicide mortality rates between the first and second lockdown in Germany in 2020.

Group	Expected suicide rate	Observed suicide rate	Expected number of suicides	Observed number of suicides	ESMR (95% CI)	Excess number of suicides (95% CI)
**Total** ^**1**^	6.4800	6.6346	5388.41	5517	1.0239 (0.9968; 1.0509)	128.59 (-16.99; 274.17)
**Men** ^**1**^	9.9235	10.0935	4071.25	4141	1.0171 (0.9862; 1.0481)	69.75 (-56.38: 195.88)
**Women** ^**1**^	3.1077	3.2662	1309.22	1376	1.0510 (0.9955; 1.1065)	66.78 (-5.93; 139.49)
**Age groups**	‐‐‐	‐‐‐	‐‐‐	‐‐‐	‐‐‐	‐‐‐
**≤24 years men** ^**1**^	1.9448	2.1202	199.96	218	1.0902 (0.9455; 1.2349)	18.04 (-10.90; 46.98)
**≤24 years women** ^**1**^	0.7167	0.9041	68.96	87	1.2616 (0.9965; 1.5267)	18.04 (-0.24; 36.32)
**25–44 years men** ^**1**^	7.3954	7.9250	787.60	844	1.0716 (0.9993; 1.1439)	56.40 (-0.54; 113.34)
**25–44 years women** ^**1**^	2.0745	2.3992	210.98	244	1.1565 (1.0114; 1.3016)	33.02 (2.40; 63.64)
**45–64 years men** ^**1**^	11.7607	11.6715	1419.76	1409	0.9924 (0.9406; 1.0442)	-10.76 (-84.33; 62.81)
**45–64 years women** ^**1**^	4.3482	4.0872	525.55	494	0.9400 (0.8571; 1.0228)	-31.55 (-75.11; 12.01)
**≥65 years men** ^**1**^	20.6748	20.8164	1658.64	1670	1.0068 (0.9586; 1.0551)	11.36 (-68.74; 91.46)
**≥65 years women** ^**1**^	4.8271	5.3761	494.74	551	1.1137 (1.0207; 1.2067)	56.26 (10.25; 102.27)

CI = confidence interval; ESMR = excess suicide mortality rate.

^1^ Prediction from a regression analysis was based on annual suicide rates for the period between the first and second lockdown between 2010 and 2019.

### Was there a change in the proportion of suicide methods in 2020 during the first lockdown compared to the previous ten years in Germany?

**Fig 2:**
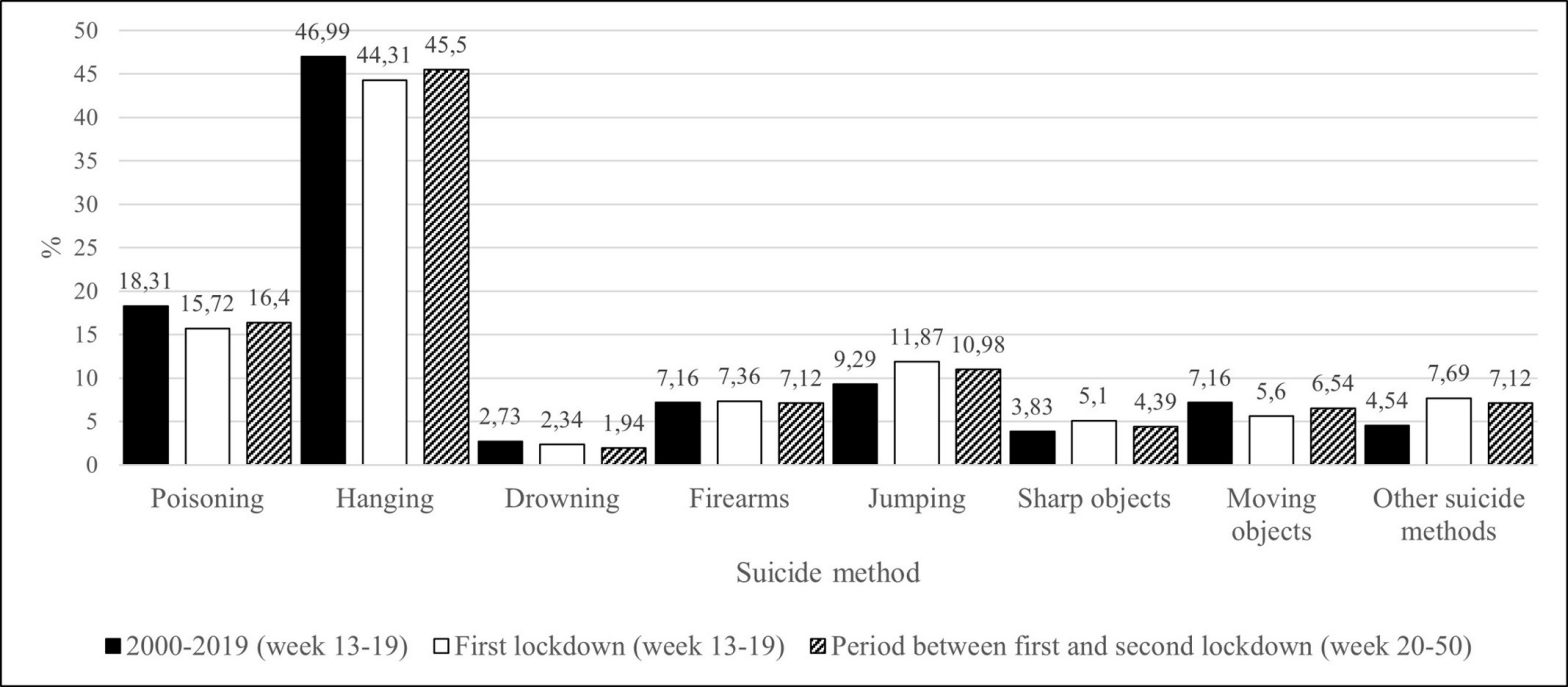
Percentage of chosen suicide methods during baseline, first lockdown, and the period between the first and the second lockdown. Suicide methods were defined as follows according to ICD-10 codes X60-X84: Self-poisoning (X60-X69), hanging (X70), drowning (X71), firearms (X72-X75), sharp objects (X78), jumping (X80), moving objects (X81,X82), other methods (X76, X77, X79, X83, X84).

Changes in the proportion of suicide methods during the first lockdown in Germany in 2020 compared to the ten-year baseline are illustrated in Fig 2.

Overall, the percentage of jumping significantly increased during the first lockdown compared to the baseline period (9.29% versus 11.87%; χ^2^ = 8.54; df = 1; p = 0.003). The same was true for ‘other methods’ (all suicide methods except for poisoning, hanging, drowning, firearms, jumping, sharp objects, and moving objects; 4.54% versus 7.69%; χ^2^ = 24.04; df = 1; p < 0.001). The percentage of poisoning significantly decreased during the first lockdown compared to the baseline period (18.31% versus 15.72%; χ^2^ = 4.98; df = 1; p = 0.026). The same was true for moving objects (7.16% versus 5.60%; χ^2^ = 4.06; df = 1; p = 0.044). For all other suicide methods, the aforementioned differences failed to be statistically significant (for details see Fig 2).

Regarding men, the percentage of jumping significantly increased during the first lockdown compared to the baseline (8.07% versus 11.09%; χ^2^ = 9.83; df = 1; p = 0.002). The same was true for the percentage of ‘other methods’ (3.96% versus 6.94%; χ^2^ = 18.25; df = 1; p < 0.001). The percentage of poisoning significantly decreased during the first lockdown compared to baseline (14.64% versus 10.97%; χ^2^ = 8.97; df = 1; p = 0.003). For all other suicide methods in men, the corresponding differences were not significant.

Regarding women, the percentage of the following suicide methods significantly increased during the first lockdown compared to the baseline period: firearms (1.26% versus 2.64%; χ^2^ = 3.93; df = 1; p = 0.047), sharp objects (3.31% versus 5.61%; χ^2^ = 4.40; df = 1; p = 0.036) and ‘other methods’ (6.22% versus 9.90%; χ^2^ = 6.21; df = 1; p = 0.013). The percentage of moving objects significantly decreased during the first lockdown compared to baseline (7.46% versus 4.29%; χ^2^ = 4.18; df = 1; p = 0.041). For all other suicide methods, the corresponding differences failed to be significant.

The excess suicide mortality rates during the first lockdown in Germany in 2020 for different suicide methods are presented in S1 Table.

## Discussion

In the present study, we analyzed suicide data for Germany of the year 2020 compared to a ten-year baseline (2010–2019).

Suicide rates from the period of the beginning of the first lockdown until the end of the year 2020 (03/22/2020-12/31/2020) did not differ significantly from the same period in the previous ten years. This is in line with other studies on the matter [12–14]. When taking the weekly suicide rates of 2020 as the basis for the analysis, we found a significant difference to the weekly distribution of suicide rates in the years before, but post-hoc analyses of the separate weeks did not yield any significant differences after correction for multiple testing. Suicide rates of 2020 were further analyzed separately for the period of the first lockdown (03/22/2020-05/06/2020) and the following period between lockdowns (05/06/2020-12/16/2020): During the first lockdown, there was a significant decrease of suicide rates compared to the previous years, even though multiple new strains related to the COVID-19 pandemic and political measures to counteract the spreading of the virus have led to worsening of mental health and thereby potentially increased suicide risk. This finding is in line with other studies [12, 25]. In the period after the first lockdown until the beginning of the second lockdown, we found no significant difference in suicide rates compared to the previous years. Still, it should not be discounted that the non-significant increase of 129 suicides after the first lockdown is a high number of deaths with many individual fates and families behind it.

One reason for this decrease of suicide rates during the first lockdown could be that the lockdown period first leads to a decrease of suicides via increased social control, less access to lethal suicide methods such as railway suicides, and less opportunities for committing suicide in homes. Our results even might suggest a general suicide preventive effect of the first lockdown because of the aforementioned changes and a state of shock in view of the global novelty of the situation.

One factor that could have influenced suicide rates during the first lockdown could be the choice of suicide methods. Indeed, our analyses have shown significant changes in the percentage of chosen suicide methods during the first lockdown compared to the ten years before. There was an increase of percentage of the methods jumping from high places and ‘other methods’ and a decrease for poisoning and lying in front of a moving object. Considering that the choice of suicide method is among others determined by the access to means, it could be speculated that the percentage of the suicide method lying in front of a moving object, e.g. railway suicides, decreased since people have less frequently left their homes during the lockdown. The same argumentation can only partly serve as an explanation for the increase of the method jumping, which might be conducted from high residential buildings as well as public sites. The interpretation of the category ‘other methods’ is challenging since it includes residual categories, but also methods that are easily accessible in a home environment (death by smoke, fire, steam, blunt object). On the basis of our data, the reflections about the changing percentages of suicide methods remain speculative, especially since information about the location of the suicide could not be obtained by the Research Data Centre for reasons of confidentiality. Furthermore, it would be preferable to include data of suicide attempts and the respective methods used (data which is not routinely collected in Germany) into the analyses in order to better understand the dynamic during lockdown times.

It also has to be kept in mind that a change in the proportion of chosen suicide methods will only fragmentarily be recognizable in data of completed suicides. As an example, an increase of the percentage of intoxications in suicidal behavior will not necessarily become visible when considering the methods used for completed suicides, since suicide attempts with this method are survived in more than 90% of cases [20]. In line with this reasoning, Reif-Leonhard and colleagues found more intoxications in suicide attempts, but not in completed suicides in the first months of the COVID-19 pandemic [22].

Limitations of our study include the possible multifactorial causes to the course of the suicide rates in the first year of the COVID-19 pandemic. We could therefore only speculate on the underlying mechanisms of the decrease of suicide rates during the lockdown, including the duration of the lockdown exposure to take effect in terms of suicidality. Inherently, our study design does not allow to conclude that there is a causal relationship between the lockdown and suicide rates. To this end, we would at least have to take into account the suicide rates of the second lockdown, which were not available at the time of the analysis.

Taken together, our data has not shown a significant change of suicide rates in 2020 overall compared to previous years. Still, the decrease of suicide rates during the first lockdown was a hint to the impact that the exceptional circumstances of the COVID-19 pandemic had had. This topic should be further investigated in order to better understand the dynamics during crises and thereby being able to derive supportive measures for people at risk of suicide.

## Supporting information

S1 Table Excess suicide mortality rates during the first lockdown in Germany in 2020 for different suicide methods. β = standardized regression coefficient for annual suicide rates; p = p value derived from a linear regression analysis. ^1^ Forecasts (predicted rates) were based on annual suicide rates for the period of the first lockdown between 2010–2019. ^2^ Due to a positive autocorrelation (d = 0.715 according to the Durbin-Watson test) a parameter estimation with robust standard errors according to the HC3 method has been computed. ^+^ p ≤ 0.10; * p ≤ 0.05; ** p ≤ 0.01; *** p ≤ 0.001.
(DOCX)
