# Supplementary material for: Suicide rates during and after the first COVID-19 lockdown in Germany in 2020
Source: PLoS One. 2023 Sep 1;18(9):e0289136. doi: 10.1371/journal.pone.0289136 (PMC10473467; doi:10.1371/journal.pone.0289136)
Supplement: S1 Table — β = standardized regression coefficient for annual suicide rates; p = p value derived from a linear regression analysis. 1 Forecasts (predicted rates) were based on annual suicide rates for the period of the first lockdown between 2010–2019. 2 Due to a positive autocorrelation (d = 0.715 according to the Durbin-Watson test) a parameter estimation with robust standard errors according to the HC3 method has been computed. + p ≤ 0.10; * p ≤ 0.05; ** p ≤ 0.01; *** p ≤ 0.001. (DOCX) [file pone.0289136.s001.docx]

**Table S1. Excess suicide mortality rates during the first lockdown in Germany in 2020 for different suicide methods**

| **Group** | **Time trend for the baseline period (2010-2019)** | **Expec-ted suicide rate** | **Ob-served suicide rate** | **Expec-ted number of suicides** | **Ob-served number of suicides** | **ESMR (95% CI)** | **Excess number of suicides (95% CI)** |
| --- | --- | --- | --- | --- | --- | --- | --- |
| **Men** | **---** | **---** | **---** | **---** | **---** | **---** | **---** |
| **Total^1^** | β=-0.672 p=0.033* | 2.3382 | 2.1766 | 959.26 | 893 | 0.9309  (0.8699; 0.9920) | -66.26  (-124.83; -7.6891) |
| **Poiso-ning** | β=-0.671 p=0.034* | 0.3249 | 0.2389 | 133.28 | 98 | 0.7353  (0.5897; 0.8809) | -35.28  (-54.68;  -15.88) |
| **Hanging** | β=-0.709 p=0.022* | 1.1613 | 1.0725 | 476.46 | 440 | 0.9235  (0.8372; 1.0098) | -36.46  (-77.57; 4.65) |
| **Drow-ning** | β=0.098 p=0.788 | 0.0478 | 0.0390 | 19.61 | 16 | 0.8159  (0.4161; 1.2157) | -3.61  (-11.45; 4.23) |
| **Fire-arms** | β=-0.311 p=0.382 | 0.2151 | 0.1950 | 88.23 | 80 | 0.9067  (0.7080; 1.1054) | -8.23  (-25.76; 9.30) |
| **Sharp objects** | β=0.290 p=0.417 | 0.1121 | 0.1072 | 46 | 44 | 0.9565  (0.6739; 1.2392) | -2.00  (-15.00; 11.00) |
| **Jumping** | β=-0.002 p=0.995 | 0.2073 | 0.2413 | 85.03 | 99 | 1.1643  (0.9349; 1.3936) | 13.97  (-5.53; 33.47) |
| **Moving objects** | β=-0.783 p= 0.007** | 0.1342 | 0.1316 | 55.06 | 54 | 0.9807  (0.7192; 1.2423) | -1.06  (-15.46; 13.34) |
| **Other suicide methods** | β=0.713 p=0.104^2^ | 0.1355 | 0.1511 | 55.59 | 62 | 1.1153  (0.8377; 1.3929) | 6.41  (-9.02; 21.84) |
| **Women** | --- | --- | --- | --- | --- | --- | --- |
| **Total^1^** | β=-0.810 p= 0.005** | 0.7631 | 0.7192 | 321.47 | 303 | 0.9425  (0.8364; 1.0487) | -18.47  (-52.59; 15.65) |
| **Poiso-ning** | β=-0.572 p=0.084^+^ | 0.2269 | 0.2136 | 95.58 | 90 | 0.9416  (0.7471; 1.1362) | -5.58  (-24.17; 13.01) |
| **Hanging** | β=-0.630 p=0.051^+^ | 0.2519 | 0.2136 | 106.11 | 90 | 0.8482  (0.6729; 1.0234) | -16.11  (-34.70; 2.48) |
| **Drow-ning** | β=-0.675 p=0.032* | 0.0278 | 0.0285 | 11.73 | 12 | 1.0230  (0.4442; 1.6018) | 0.27  (-6.52; 7.06) |
| **Fire-arms** | β=0.104 p=0.774 | 0.0115 | 0.0190 | 4.86 | 8 | 1.6461  (0.5054; 2.7868) | 3.14  (-2.40; 8.68) |
| **Sharp objects** | β=-0.500 p=0.141 | 0.0212 | 0.0404 | 8.92 | 17 | 1.9058  (0.9999; 2.8118) | 8.08  (-0.001; 16.16) |
| **Jumping** | β=-0.679 p=0.031* | 0.0952 | 0.1021 | 40.11 | 43 | 1.0721  (0.7516; 1.3925) | 2.89  (-9.96; 15.74) |
| **Moving objects** | β=-0.514 p=0.129 | 0.0432 | 0.0309 | 18.19 | 13 | 0.7147  (0.3262; 1.1032) | -5.19  (-12.26; 1.88) |
| **Other suicide methods** | β=0.878 p= 0.001*** | 0.0854 | 0.0712 | 35.97 | 30 | 0.8340  (0.5356; 1.1325) | -5.97  (-16.71; 4.77) |

**Notes:** β = standardized regression coefficient for annual suicide rates; p = p value derived from a linear regression analysis.

^1^ Forecasts were based on annual suicide rates for the period of the first lockdown between 2010-2019.

^2^ Due to a positive autocorrelation (d = 0.715 according to the Durbin-Watson test) a parameter estimation with robust standard errors according to the HC3 method has been computed.

^+^ p ≤ 0.10; * p ≤ 0.05; ** p ≤ 0.01; *** p ≤ 0.001.
